# Spatial Transcriptomics Unveils Landscape of Resistance to Concurrent Chemo‐Radiotherapy in Hypopharyngeal Squamous Cell Carcinoma: The Role of *SPP1* ^*+*^ Macrophages

**DOI:** 10.1002/cam4.71493

**Published:** 2025-12-29

**Authors:** Jungyoon Ohn, Sungwoo Bae, Hongyoon Choi, Jiwon Koh, In Gul Kim, Eun‐Jae Chung, Kwon Joong Na

**Affiliations:** ^1^ Portrai, Inc. Seoul Republic of Korea; ^2^ Department of Nuclear Medicine Seoul National University Hospital Seoul Republic of Korea; ^3^ Department of Pathology Seoul National University Hospital Seoul Republic of Korea; ^4^ Department of Otolaryngology‐Head and Neck Surgery, Biomedical Research Institute Seoul National University Hospital Seoul Republic of Korea; ^5^ Department of Otolaryngology‐Head and Neck Surgery Seoul National University College of Medicine Seoul Republic of Korea; ^6^ Department of Thoracic and Cardiovascular Surgery Seoul National University Hospital Seoul Republic of Korea

**Keywords:** concurrent chemoradiotherapy resistance, hypopharyngeal squamous cell carcinoma, macrophage, secreted phosphoprotein 1, spatial transcriptomics, tumor microenvironment

## Abstract

**Background:**

Hypopharyngeal squamous cell carcinoma (SCC) is an aggressive malignancy with a poor prognosis, particularly in advanced stages. Concurrent chemoradiotherapy (CCRT) is frequently employed to preserve the larynx, but resistance to CCRT remains a significant clinical challenge. Understanding the tumor microenvironment (TME) in CCRT‐resistant cases is crucial for identifying predictive biomarkers and developing targeted therapies to improve outcomes.

**Methods:**

This study analyzed tissue samples from patients with advanced hypopharyngeal SCC who were either resistant to CCRT or had not received CCRT. Spatial transcriptomics (ST) was used to explore the spatial molecular signatures within the TME of these samples, focusing on the interactions between immune cells and malignant cells.

**Results:**

We identified six distinct cellular clusters in the hypopharyngeal SCC tissues, with a signature cluster more prominently present in CCRT‐resistant samples. The SPP1 gene was significantly overexpressed in these samples, specifically in macrophages, and was associated with increased ligand–receptor interactions involving malignant cells via CD44 and ITGB1. These interactions were primarily observed in peri‐tumoral and intratumoral regions, indicating a role for SPP1+ macrophages in modulating the TME and contributing to CCRT resistance. Further analysis revealed that SPP1‐mediated cell–cell interactions predominantly occurred between macrophages and malignant epithelial cells, highlighting their potential role in driving therapeutic resistance.

**Conclusions:**

Our findings suggest that SPP1‐expressing macrophages play a pivotal role in the development of CCRT resistance in hypopharyngeal SCC through specific interactions with malignant cells. The spatial distribution of these macrophages and their interaction with cancer cells suggest a mechanism by which the TME contributes to therapeutic failure. These insights could inform the development of novel targeted therapies aimed at overcoming CCRT resistance, ultimately improving patient outcomes.

**Abbreviations**
CCRT: concurrent chemoradiotherapy
LNM: lymph node metastasis
SCC: squamous cell carcinoma
ST: spatial transcriptomics
TME: tumor microenvironment
UMAP: uniform manifold approximation and projection

## 1 Introduction

Hypopharyngeal squamous cell carcinoma (SCC) is a highly aggressive cancer often diagnosed at an advanced stage, with many cases involving lymph node metastasis, leading to a notoriously poor prognosis [1]. Regardless of the poor prognosis, the most critical issue for patients and caregivers in the treatment of hypopharyngeal SCC is whether the larynx can be preserved to maintain normal breathing, speaking, and swallowing functions. Laryngeal preservation, such as partial pharyngolaryngectomies, through surgical treatment, is only possible in early‐stage cancer. Advanced tumors of the hypopharynx (over T3) are mostly managed by total laryngectomy followed by radiation therapy or concurrent chemoradiotherapy (CCRT), called the organ preservation protocol, depending on the patient's overall health status and surgical feasibility [1]. The organ preservation protocol can preserve the larynx, allowing patients to maintain natural speech and respiratory function, which may improve quality of life [1]. However, there is currently no method to predict the CCRT resistance in the individual patient [2, 3].

Despite the established efficacy of CCRT, the clinical landscape is frequently marred by the emergence of CCRT resistance, precipitating therapeutic failure and progression of disease. Critically, CCRT exerts multifaceted influences on the immune and stromal cellular constituents of the tumor microenvironment (TME): immunomodulation, re‐vascularization, inflammation, extracellular matrix remodeling by cancer‐associated fibroblasts, and fibrosis [4]. This modulation of the TME can subsequently alter the biological aggressiveness of the tumor, underscoring the complexity of treatment response and resistance in hypopharyngeal SCC. Tumor‐associated macrophages (TAMs), exhibiting remarkable plasticity and polarization states ranging from pro‐inflammatory M1‐like to immunosuppressive M2‐like phenotypes, play pivotal roles in shaping the TME [5, 6]. Their dynamic interactions with tumor and stromal cells influence angiogenesis, extracellular matrix remodeling, and immune evasion, thereby contributing to tumor progression and treatment resistance. In hypopharyngeal SCC, macrophage infiltration has been linked to poor prognosis, underscoring the need to better understand their functional roles, particularly in the context of CCRT resistance.

Consequently, to surmount CCRT resistance and identify novel biomarkers predictive of such resistance, a profound understanding of the TME dynamics under these therapeutic conditions is paramount [4]. This study aims to explore the TME of hypopharyngeal SCC, which recurs after CCRT, with the objective of identifying spatial molecular signatures within the cancer tissue using spatial transcriptomics (ST). By employing ST, we seek to gain insights into the spatial distribution of transcriptomic changes associated with CCRT resistance in hypopharyngeal SCC. Such insights might hold promise for the development of targeted therapeutic strategies and improved patient management in CCRT‐resistant hypopharyngeal SCC.

## 2 Materials and Methods

### 2.1 Patient Enrollment

**FIGURE 1:**
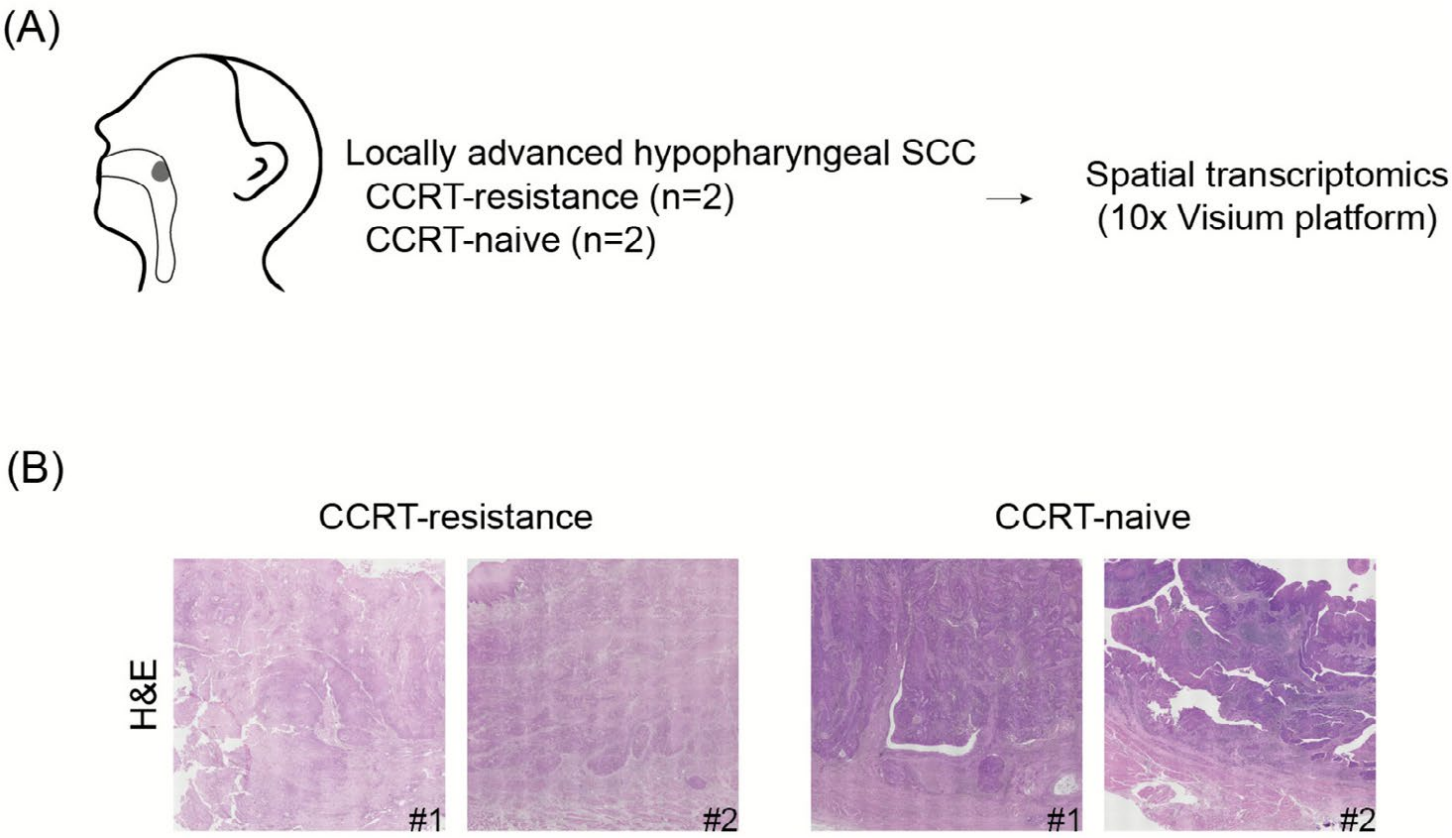
Spatial profiling for patients with hypopharyngeal SCC. (A) Schematic of study (*n* = 2, CCRT‐resistance; *n* = 2, CCRT‐naive). (B) H&E‐stained tissue slides. Abbreviations: CCRT, concurrent chemo‐radiotherapy; SCC, squamous cell carcinoma.

The patients with locally advanced hypopharyngeal SCC were retrospectively enrolled from the cancer tissue bank. We obtained tissue samples from two patients with locally advanced hypopharyngeal SCC who had definite CCRT as a primary treatment and total laryngectomy for recurrence of hypopharyngeal SCC (designated as CCRT‐resistance) and from two patients with locally advanced hypopharyngeal SCC who underwent upfront surgical treatment of hypopharyngeal SCC without previous CCRT (designated as CCRT‐naive) (Figure 1A). Detailed clinical information of all study patients was shown in Supporting Table. The clinical study was approved by the institutional review board of Seoul National University Hospital (approval date: 27/12/2023, approval number: H‐2203‐011‐1304) as a minimal‐risk retrospective study, and the individual consent was waived.

### 2.2 Sample Acquisition and *p16* Immunohistochemistry

Formalin‐fixed paraffin‐embedded (FFPE) blocks from the surgical specimen were used for p16 immunohistochemistry (IHC). FFPE blocks were cut into 4 μm sections, and we used anti‐p16 mouse monoclonal antibody (clone E6H4; Ventana Medical Systems, Tucson, AZ, USA) for IHC using the Ventana Benchmark XT automated immunostainer (Ventana Medical Systems). p16 IHC was interpreted by a head and neck pathologist (J.K.), where p16 positivity was defined as strong, diffuse nuclear and cytoplasmic staining in ≥ 70% of the tumor cells [7].

### 2.3 Sample Sequencing: Visium ST

Tumor tissues were obtained from the surgical specimen, fixed with formalin, and embedded in paraffin blocks for preservation. To process FFPE samples for RNA sequencing, the paraffin blocks were sectioned, mounted on glass slides, and deparaffinized. The tissue sections were stained with hematoxylin–eosin (H&E) and de‐crosslinked. After the hybridization of the probes that capture the specific sequence of RNA, the Visium CytAssist device (10x Genomics, USA) was utilized to transfer the tissue onto the Visium slide. The tissue was permeabilized, RNAs were captured, reverse‐transcribed into cDNA, and amplified to construct gene expression libraries. The libraries were sequenced, and the raw sequence files were processed from BCL to FASTQ format and then to count matrices using Space Ranger (10x Genomics, USA) custom pipeline. H&E‐stained images were aligned with the RNA capture plane and processed for downstream analysis.

### 2.4 Preprocessing ST Datasets

To remove the low‐quality data in Visium ST, spots with a number of genes with non‐zero expression greater than 500 and a percentage of mitochondrial genes lower than 20% were selected. The raw count matrix for each Visium slide was normalized and scaled using *SCTransform* [8]. *SCTransform* transforms the raw count of the gene to Pearson residuals based on regularized negative binomial regression, with the total count and percentage of mitochondrial genes as covariates. Canonical correlation analysis (CCA) was performed to embed spots from pairs of slides into low low‐dimensional space, and mutual nearest neighbors (MNN) were found between the slides to correct the batch effect [9]. After batch correction, the dimensionality was reduced using principal component analysis (PCA), and 30 dimensions (PC1–PC30) were chosen for the clustering of spots using the Louvain algorithm (resolution = 0.15) [10]. The markers of each spot cluster were discovered using the Wilcoxon rank‐sum test, and the Bonferroni method was applied for multiple comparison corrections. The spot clusters were named according to the signature gene sets composed of the top 20 markers (Supplementary File). All of the above analyses were performed in *Seurat* (version 4.4.0) running on R [9] and *scanpy* (version 1.10.1) running on Python [11].

### 2.5 Estimation of Cell Type Distribution in the TME

The abundance of cells in each spot was estimated using *Cell2location* (version 0.1.4) [12]. *Cell2location* uses a reference single‐cell transcriptomics dataset and applies a negative binomial regression model to extract gene expression profiles of cell types in the tumor. A publicly available single‐cell RNA sequencing dataset obtained from 20 hypopharyngeal SCCs (accession number GSE181919) and cell type annotation information were used to infer the cell type signatures of nine cell types: cancer cells, endothelial cells, fibroblasts, myocytes, macrophages, dendritic cells, mast cells, plasma cells, and T cells [13]. For deconvolution, we applied Cell2location (v0.1.4) with the following parameters: the expected average number of cells per Visium spot (*N_cells_per_location*) was set to 10, and the detection alpha (*detection_alpha*) was set to 20, as recommended by the developers. Then, leveraging cell type signatures, the spatial abundance of cell types and cell type‐specific expression profiles at each spot location were predicted.

### 2.6 Annotation of the Cancer Region and Calculation of Distance From the Cancer

Pathological annotation of the dataset was performed by a board‐certified pathologist. The tumor tissues were divided into nine categories: “SqCC,” cancer regions, including invasive carcinoma and carcinoma in situ; “Lym (+) stroma,” stroma with immune cell‐abundant; “Lym (−) stroma,” immune cell‐depleted stroma; “Glandular stroma,” gland‐rich regions; “Blood vessels,” blood vessels regions; “Anucleated keratin,” regions with keratin materials; “Normal mucosa,” normal squamous mucosa; “Muscle,” smooth muscle regions; and “Necrosis,” necrotic regions. Notably, CCRT‐resistance samples had low quality of the H&E stain, and the pathologic annotation was performed at the level of differentiating tumoral and stromal compartments.

To complement the pathological annotation for the low‐quality H&E images and double‐check the cancer region within the slide, we applied a deep learning model, *Cancer‐Finder* [14]. *Cancer‐Finder* was trained on ST datasets of several cancer types and classifies the spots into noncancer and cancer spots based on transcriptomic profiles. After the segmentation of the cancer region, the annotation results were compared between those from pathologists and from transcriptomic profiles.

The physical distance from the annotated cancer region to a certain spot was defined using the function *annotation_coordinate* in the package *tacco* (version 0.01‐post2) [15]. The distance between the spots was calculated by assuming the spots are placed on the hexagonal coordinates and by setting the size of the bin to 1. The estimated distance was used to evaluate the spatial patterns of genes, cell types, and ligand–receptor (LR) interactions with respect to the cancer region.

### 2.7 LR Interaction Analysis

The spatial co‐expression pattern between ligand and receptor was captured using a method called *stLearn* (version 0.4.12) [16]. The strength of LR interaction in the Visium slide was calculated by averaging the mean ligand expression in the spot and its surrounding neighbors expressing the receptor and vice versa. The scores were calculated for all LR pairs in the Omnipath database, and the top LR pairs were ranked [17]. The significance of the interaction was computed based on a random background distribution derived from 10,000 noninteracting gene pairs with similar expression levels. In addition, using the cell type abundance estimated by *Cell2location*, the cell type‐specific interaction patterns were investigated. The spots were annotated by the most abundant cell type in each location, and the number of neighboring spots that express the ligand or receptor was counted. The cell labels were permutated 500 times while maintaining the cell type proportion in the whole slide to create a random distribution. Then, the probability that a certain LR interaction occurs between cell type pairs was calculated. The resulting LR interactions between cell type pairs were presented with a cell–cell interaction network plot.

## 3 Results

### 3.1 ST Reveals Distinct Cellular Clusters in Hypopharyngeal SCCs Tissues

**FIGURE 2:**
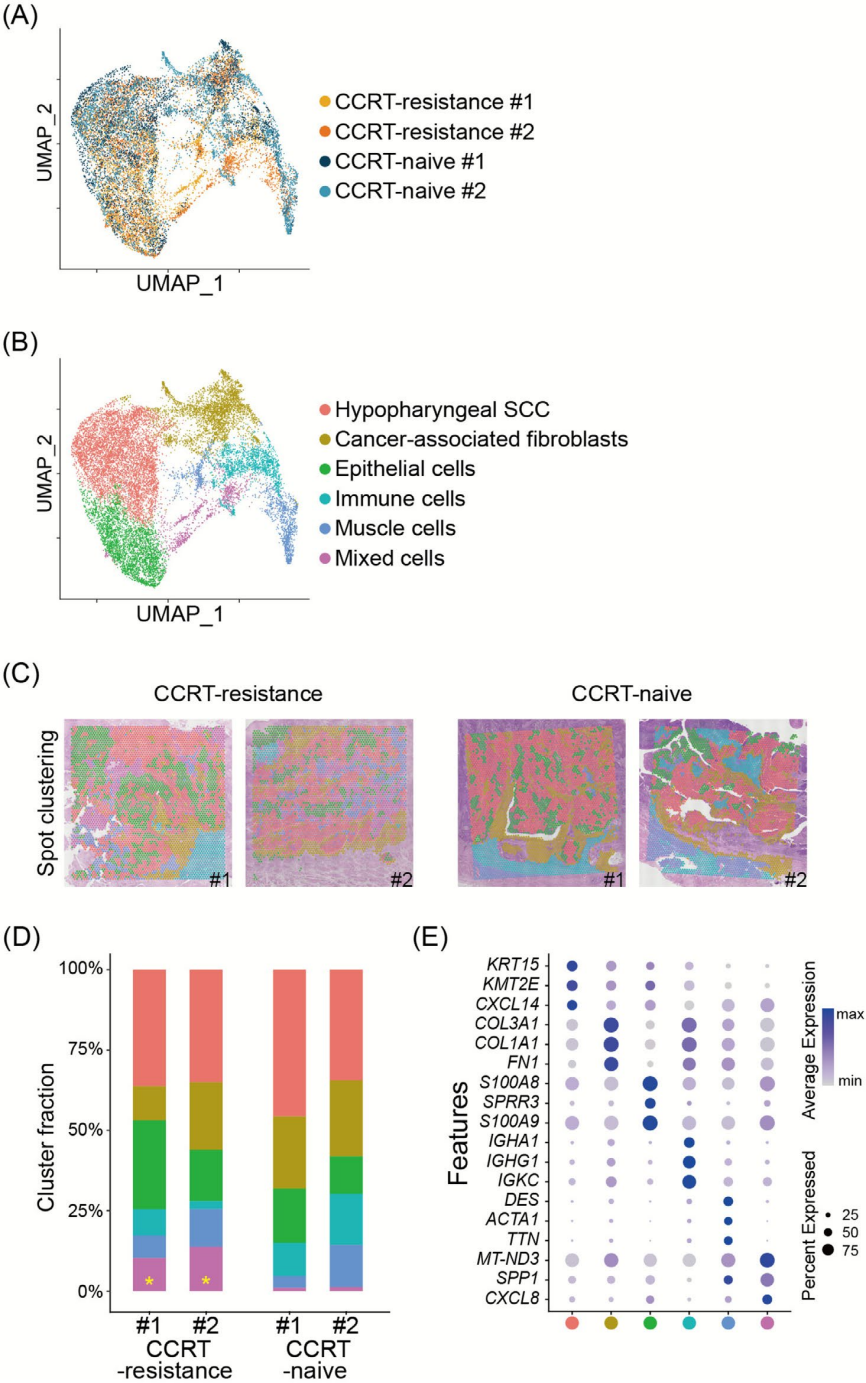
Distinct spot clusters identified by ST analysis of hypopharyngeal SCC samples. (A) UMAP of ST spots color‐coded by sample origin. (B) UMAP of ST spots color‐coded by cluster identities defined based on their signature gene sets. (C) Spatial location of the spot clusters. (D) The fraction of the number of ST spots corresponding to the spot cluster in each sample. The yellow asterisk indicates a significant cluster in the CCRT‐resistant group. (E) The top three signature genes of each cluster (selected with a log fold change threshold and minimum expression percentage both set to 0.25). Abbreviations: CCRT, concurrent chemo‐radiotherapy; SCC, squamous cell carcinoma; UMAP: Uniform manifold approximation and projection.

In both the CCRT‐resistant and naive groups, H&E images showed the presence of hypopharyngeal SCC, characterized by abnormal squamous epithelial proliferation, nuclear pleomorphism, increased mitotic activity, or keratinization (Figure 1B). Upon plotting the obtained ST data spots on the uniform manifold approximation and projection for dimension reduction (UMAP) plot, the spots from the four different tissues showed significant overlap, implying minimized batch effects (Figure 2A). In addition, these spots clustered into six distinct groups based on key gene expression identifiers (Figure 2B,C, Figure S1, and Supporting File): Hypopharyngeal scc, cancer‐associated fibroblasts, epithelial cells, immune cells, mixed cell areas.

### 3.2 Secreted Phosphoprotein 1 (SPP1) as a Key Marker in CCRT‐Resistant Hypopharyngeal SCC

**FIGURE 3:**
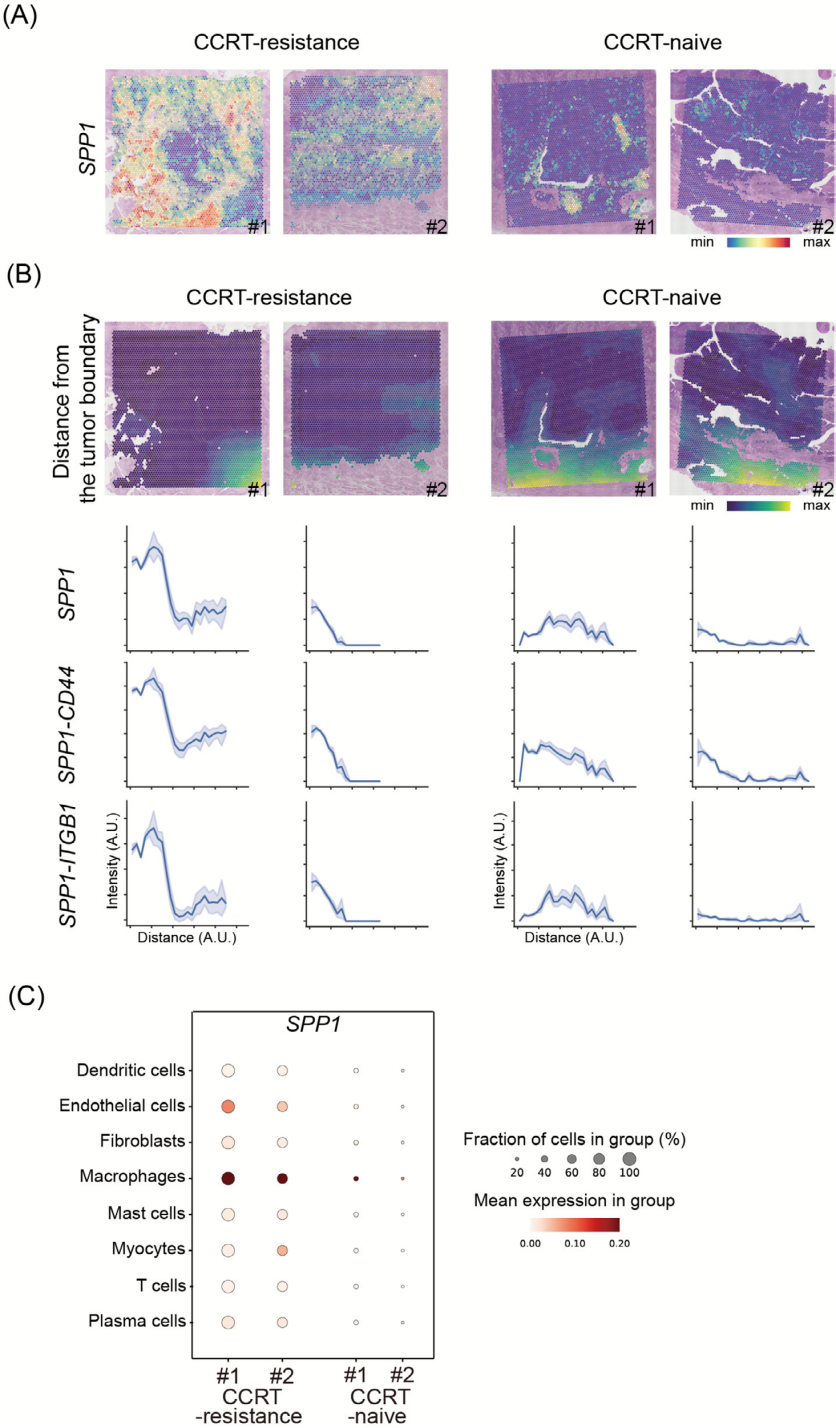
Intra and peri‐tumoral *SPP1+* macrophages are associated with CCRT‐resistant hypopharyngeal SCC. (A) The distribution pattern of *SPP1* gene expression for each sample overlaid on the H&E slide. (B) Relative spatial distance from the tumor boundary and corresponding *SPP1* expression, *SPP1–CD44*, and *SPP1–ITGB1* interaction strength. (C) Estimated *SPP1* expression levels in each cell type within the TME. Abbreviations: CCRT, concurrent chemo‐radiotherapy; SCC, squamous cell carcinoma.

Interestingly, the mixed cell area was more prominent in the CCRT‐resistant tissue samples compared to the CCRT‐naïve tissue samples (Figure 2D). *SPP1* was identified as a signature gene with the highest differential expression between the mixed cell cluster and other clusters (Figure 2E). In line with these findings, when examining the expression pattern of the *SPP1* gene across the whole tissue, it was notably elevated in the CCRT‐resistant TME compared to the CCRT‐naïve group (Figure 3A). Along with the observation of high expression of *SPP1* in the CCRT‐resistant tissue samples, LR interaction analysis using *stLearn* was performed on each tissue sample, revealing the top 50 LR interactions, respectively (Figure S2). Among the interactions, *SPP1* ligand‐mediated interactions (*SPP1–CD44* and *SPP1–ITGB1*) were commonly found in the CCRT‐resistant group; however, they were not observed in the CCRT‐naïve group.

### 3.3 *SPP1* Ligand‐Mediated Interactions Were Detected in TME

Next, we explored the spatial distribution of the *SPP1–CD44* and *SPP1–ITGB1* interactions within TME. To accurately characterize the histological architecture of each hypopharyngeal SCC into cancer or surrounding stroma in TME, the *Cancer‐Finder* tool was used [14]. Also, the histopathology of H&E‐stained slide images was reviewed by a board‐certified pathologist (Figure S3). We found that these interactions were primarily detected in the peri‐tumoral spatial region, as well as in the intratumoral spatial area (Figure 3B and Figure S4).

### 3.4 Macrophage‐Specific *SPP1* Expression and Cell–Cell Interactions in CCRT‐Resistant Hypopharyngeal SCC

To further investigate which cells within the diverse cell populations constituting the hypopharyngeal SCC TME predominantly express the *SPP1* gene, we utilized the bioinformatic tool *Cell2location*. The expression level of SPP1 is elevated, particularly in macrophages, among various cell types (Figure 3C and Figure S5). Then, we aimed to identify the *SPP1*‐mediated cell–cell interaction pairs involving *SPP1* ^+^ macrophages in CCRT‐resistant hypopharyngeal SCC tissues using the *stLearn* tool. The analysis showed a higher number of interactions from macrophages to malignant epithelial cells in the CCRT‐resistant tissues compared to the CCRT‐naïve tissues. In CCRT‐resistant tissues, *SPP1*–*CD44* and *SPP1*–*ITGB1* interactions occur primarily between malignant epithelial cells and macrophages, while these interactions occur mainly between macrophages and endothelial cells or fibroblasts in CCRT‐naive samples. The conspicuous absence of macrophage‐to‐malignant cell interactions in the CCRT‐naive group was noteworthy (Figure S6).

## 4 Discussion

Herein, it was found that CCRT‐resistant hypopharyngeal SCC was characterized by *SPP1* ^+^ macrophages in TME, which interact with malignant epithelial cells in intratumoral and peri‐tumoral regions via *SPP1–CD44* and *SPP1–ITGB1*. The *SPP1* gene encodes SPP1 protein, also known as osteopontin. *SPP1* is a multifunctional integrin‐binding glycoprotein, which is overexpressed in a variety of tumors, playing a pivotal role in controlling the cell signaling pathway for tumor progression and metastasis [18].

Notably, the *SPP1* gene has been identified as a significant biomarker in hypopharyngeal SCC [19] with its upregulated mRNA expression in tumor tissues correlating with poor overall survival of hypopharyngeal SCC patients [20] Additionally, its role in promoting lymph node metastasis (LNM) in hypopharyngeal SCC has been proposed [21] Analysis of large public datasets [22] suggested the role of interactions, *SPP1–CD44* and *SPP1–ITGB1*, in hypopharyngeal SCC progression. *CD44* has also been associated with worse tumor grade/staging and prognosis in pharyngeal and laryngeal cancers [23]. *CD44* promotes tumor progression by enhancing cell migration, invasion, and metastasis. It is a key cancer stem cell marker and contributes to tumor aggressiveness through epithelial–mesenchymal transition and signaling pathways [23]. Patients with hypopharyngeal SCC who exhibit concurrent expression of integrin beta 1 (ITGB1) and neurogenic locus notch homolog protein 1 (NOTCH 1) protein experience significantly poorer survival rates, potentially through the regulation of cancer stemness [24].

Of particular note, *SPP1*+ TAM infiltration is associated with LNM and poor prognosis in hypopharyngeal SCC patients [25]. Also, macrophages defined by *SPP1* and *CXCL9* expression are strongly associated with hypopharyngeal SCC prognosis [26]. The infiltration of *SPP1* ^+^ macrophages gradually increases with tumor progression, and their interaction with cancer cells gradually increases to reprogram malignant cells, leading to tumor upstaging and shaping of the desmoplastic microenvironment [27]. Our findings of enriched *SPP1* ^+^ macrophages in CCRT‐resistant hypopharyngeal SCC align with the broader concept of macrophage plasticity and polarization within the TME. TAMs are not static entities but exist along a continuum between M1‐like and M2‐like states, dynamically adapting to tumor‐derived and stromal signals. Such plasticity enables TAMs to orchestrate extracellular matrix remodeling, angiogenesis, and immune suppression, thereby fostering tumor progression and therapeutic resistance [5, 6]. In this context, *SPP1* ^+^ macrophages may represent a specialized TAM subset with potent tumor‐promoting capacity, consistent with prior reports linking macrophage heterogeneity to cancer aggressiveness.

These precedent findings provide strong support for our analysis results. However, since the ST captures RNA expression in the unit of spot, which consists of a mixture of cells, further validation at the single‐cell and protein levels is required in future studies. Through this study, we have identified characteristics of TME in patients experiencing recurrence after CCRT treatment, showing heightened interaction between *SPP1*‐positive macrophages and cancer cells via *CD44* or *ITGB1*. Understanding the spatial distribution and functional implications of *SPP1*‐expressing macrophages interacting with cancer cells within the TME could provide valuable insights into the mechanisms underlying CCRT resistance in hypopharyngeal SCC. This may ultimately help to improve clinical decision‐making, guiding the development of more effective therapeutic strategies for hypopharyngeal SCC patients. A limitation of this study is the small sample size of hypopharyngeal cancer patients. Further studies with larger cohorts are necessary to validate these results.

This study is limited by the absence of CCRT‐responsive hypopharyngeal SCC samples, as such specimens are rarely available in clinical practice because patients with a complete response to CCRT typically do not undergo surgical resection. Additional limitations include the lack of paired single‐cell data for ST validation, which reduces the resolution in precisely determining the localization of gene expression within the tissue, and the small sample size, which precludes meaningful correlation of *SPP1* expression with clinical outcomes. We partially addressed the absence of paired single‐cell data by incorporating a publicly available single‐cell dataset from hypopharyngeal SCC, although treatment status information was unavailable. Despite these constraints, our findings provide an initial framework for characterizing the TME in CCRT‐resistant hypopharyngeal SCC cases. Future work will focus on collecting additional CCRT‐resistant tissues and conducting functional studies to evaluate whether blocking the *SPP1–CD44* axis could alter CCRT sensitivity.

## 5 Conclusions

We find elevated interactions between *SPP1* ^+^ macrophages and cancer cells in CCRT‐resistant tumors. These results highlight the significant role of *SPP1* ^+^ macrophages in CCRT resistance, offering potential pathways for targeted therapies and enhanced patient management in hypopharyngeal SCC.

## Author Contributions

Jungyoon Ohn: writing – original draft; writing – review and editing; formal analysis; investigation; visualization. Sungwoo Bae: writing – review and editing; writing – original draft; investigation; formal analysis; methodology. Hongyoon Choi: conceptualization; writing – review and editing; data curation; formal analysis; methodology. Jiwon Koh: methodology; data curation; investigation; writing – review and editing. In Gul Kim: conceptualization; data curation; investigation; funding acquisition. Eun‐Jae Chung: conceptualization; writing – review and editing; data curation; resources; supervision; funding acquisition. Kwon Joong Na: conceptualization; writing – original draft; writing – review and editing; supervision; funding acquisition.

## Funding

This work was supported by the Electronics and Telecommunications Research Institute (24YK1110), Seoul National University Hospital (2620210050), and National Research Foundation of Korea (2020M3A9B6037195, RS‐2024‐00357094).

## Ethics Statement

The study protocol was reviewed by the Institutional Review Board of Seoul National University Hospital, approved as a minimal‐risk retrospective study (approval date: 27/12/2023, approval number: H‐2203‐011‐1304), and individual consent was waived.

## Conflicts of Interest

H.C. and K.J.N. are the co‐founders and shareholders of Portrai Inc.

## Supporting information


**Figure S1:** Heatmap representation of marker gene expression in each spot cluster derived from ST data. Each row corresponds to a specific gene, while each column corresponds to a cluster within hypopharyngeal SCC tissue samples. Gene expression levels are depicted by color intensity, ranging from magenta (low expression) to yellow (high expression). Clusters are annotated based on the key gene expression identifiers.
Abbreviation: SCC, squamous cell carcinoma; ST, spatial transcriptomics.
**Figure S2:** The top 50 LR interaction pairs were identified using the stLearn tool, which incorporates spatial location and co‐expression in the ST data to infer the number of spots with significant interactions. In CCRT‐resistance samples, SPP1 showed a high interaction with CD44 and ITGB1 (indicated by red arrow).
Abbreviation: CCRT, concurrent chemo‐radiotherapy; LR, ligand‐receptor; ST, spatial transcriptome.
**Figure S3:** Annotation of cancerous and noncancerous regions. (A) Application of the Cancer‐Finder algorithm to four hypopharyngeal SCC ST datasets. (B) Annotation by a board‐certified pathologist. The annotated spots were overlaid on the H&E‐stained slides (refer to Annotation of the cancer region and calculation of distance from the cancer in MATERIALS AND METHODS).
Abbreviation: SCC, squamous cell carcinoma; ST, spatial transcriptomics.
**Figure S4:** Spatial heatmap for indexes of LR interactions for SPP1–CD44 (A) and SPP1–ITGB1 (B), overlaid on an H&E‐stained slide image in each sample. “lr_scores” represent the strength of ligand‐receptor colocalization, “p_vals” indicate the significance of “lr_scores” determined by creating random distribution of noninteracting gene–gene pairs, and “p_adjs” show the adjusted p‐values.
Abbreviation: LR, ligand‐receptor.
**Figure S5:** Heatmaps showing the abundance of malignant epithelial cells and other cell types comprising the TME (dendritic cells, endothelial cells, fibroblasts, macrophages, mast cells, myocytes, T cells, and plasma cells) on ST spots for each hypopharyngeal SCC sample (top row of each panel). The bottom row of each panel illustrates the spatial expression of SPP1 and cell type‐specific SPP1 expression in four hypopharyngeal SCC samples. This figure was generated using the Cell2location package.
Abbreviation: CCRT, concurrent chemo‐radiotherapy; SCC, squamous cell carcinoma; ST, spatial transcriptomics; TME, tumor microenvironment.
**Figure S6:** In each study sample, cell–cell interactions were analyzed to identify predominant cells involved in LR interactions, SPP1–CD44 (A) and SPP1–ITGB1 (B). The size of the spots represents the total number of significant spot interactions the cell type is involved in, and the color of the edge represents the number of significant interactions between the two cell types. In CCRT‐resistance samples, SPP1–CD44 and SPP1–ITGB1 interactions occur primarily between malignant epithelial cells and macrophages, while these interactions occur mainly between macrophages and endothelial cells or fibroblasts in CCRT‐naive samples.
Abbreviation: CCRT, concurrent chemo‐radiotherapy; LR, ligand–receptor.
Supporting Table. The clinical characteristics of the patients.

**Supporting File**. Top 20 gene markers for the annotation of each spot cluster.

## Data Availability

The data that support the findings of this study are available on request from the corresponding author. The data are not publicly available due to privacy or ethical restrictions.
