# Supplementary material for: Spatial Transcriptomics Unveils Landscape of Resistance to Concurrent Chemo‐Radiotherapy in Hypopharyngeal Squamous Cell Carcinoma: The Role of SPP1 + Macrophages
Source: Cancer Med. 2025 Dec 29;15(1):e71493. doi: 10.1002/cam4.71493 (PMC12748936; doi:10.1002/cam4.71493)
Supplement: Supplementary file 1 — Figure S1: Heatmap representation of marker gene expression in each spot cluster derived from ST data. Each row corresponds to a specific gene, while each column corresponds to a cluster within hypopharyngeal SCC tissue samples. Gene expression levels are depicted by color intensity, ranging from magenta (low expression) to yellow (high expression). Clusters are annotated based on the key gene expression identifiers. Abbreviation: SCC, squamous cell carcinoma; ST, spatial transcriptomics. Figure S2: The top 50 LR interaction pairs were identified using the stLearn tool, which incorporates spatial location and co‐expression in the ST data to infer the number of spots with significant interactions. In CCRT‐resistance samples, SPP1 showed a high interaction with CD44 and ITGB1 (indicated by red arrow). Abbreviation: CCRT, concurrent chemo‐radiotherapy; LR, ligand‐receptor; ST, spatial transcriptome. Figure S3: Annotation of cancerous and noncancerous regions. (A) Application of the Cancer‐Finder algorithm to four hypopharyngeal SCC ST datasets. (B) Annotation by a board‐certified pathologist. The annotated spots were overlaid on the H&E‐stained slides (refer to Annotation of the cancer region and calculation of distance from the cancer in MATERIALS AND METHODS). Abbreviation: SCC, squamous cell carcinoma; ST, spatial transcriptomics. Figure S4: Spatial heatmap for indexes of LR interactions for SPP1–CD44 (A) and SPP1–ITGB1 (B), overlaid on an H&E‐stained slide image in each sample. “lr_scores” represent the strength of ligand‐receptor colocalization, “p_vals” indicate the significance of “lr_scores” determined by creating random distribution of noninteracting gene–gene pairs, and “p_adjs” show the adjusted p‐values. Abbreviation: LR, ligand‐receptor. Figure S5: Heatmaps showing the abundance of malignant epithelial cells and other cell types comprising the TME (dendritic cells, endothelial cells, fibroblasts, macrophages, mast cells, myocytes, T cells, and plasma cells [file CAM4-15-e71493-s001.docx]

Supplementary Table. The clinical characteristics of the patients

|  | Age | Sex | CCRT regimen | T stage | N stage | Stage^#^ | *p16*  IHC staining |
| --- | --- | --- | --- | --- | --- | --- | --- |
| CCRT-resistance #1 | 58 | Male | Weekly CDDP + RT | 2 | 2c | 4A | Negative |
| CCRT-resistance #2 | 60 | Male | Weekly CDDP + RT | 4a | 3b | 4B | Negative |
| CCRT-naïve #1 | 60 | Male | NA | 3 | 3b | 4A | Negative |
| CCRT-naïve #2 | 72 | Female | NA | 3 | 3b | 4B | Negative |

^#^Clinical stage was based on AJCC 7^th^ edition.

Abbreviation: CCRT, concurrent chemo-radiotherapy; CDDP, cisplatin; IHC, immunohistochemistry; NA, not applicable; RT, radiotherapy

.

Supplementary Figures


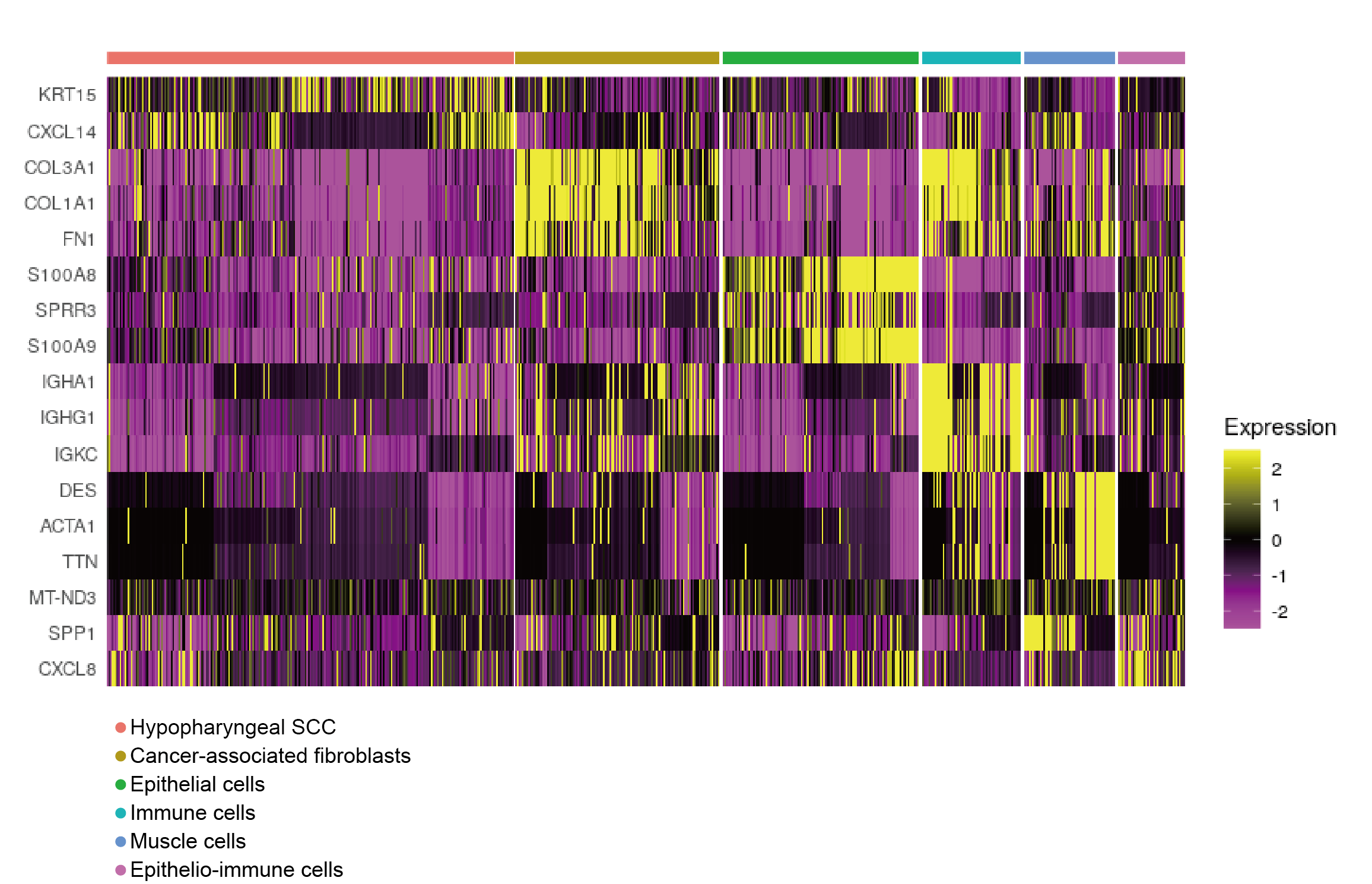


Supplementary Figure 1. Heatmap representation of marker gene expression in each spot cluster derived from ST data. Each row corresponds to a specific gene, while each column corresponds to a cluster within hypophayngeal SCC tissue samples. Gene expression levels are depicted by color intensity ranging from magenta (low expression) to yellow (high expression). Clusters are annotated based on the key gene expression identifiers.

Abbreviation: SCC, squamous cell carcinoma; ST, spatial transcriptomics


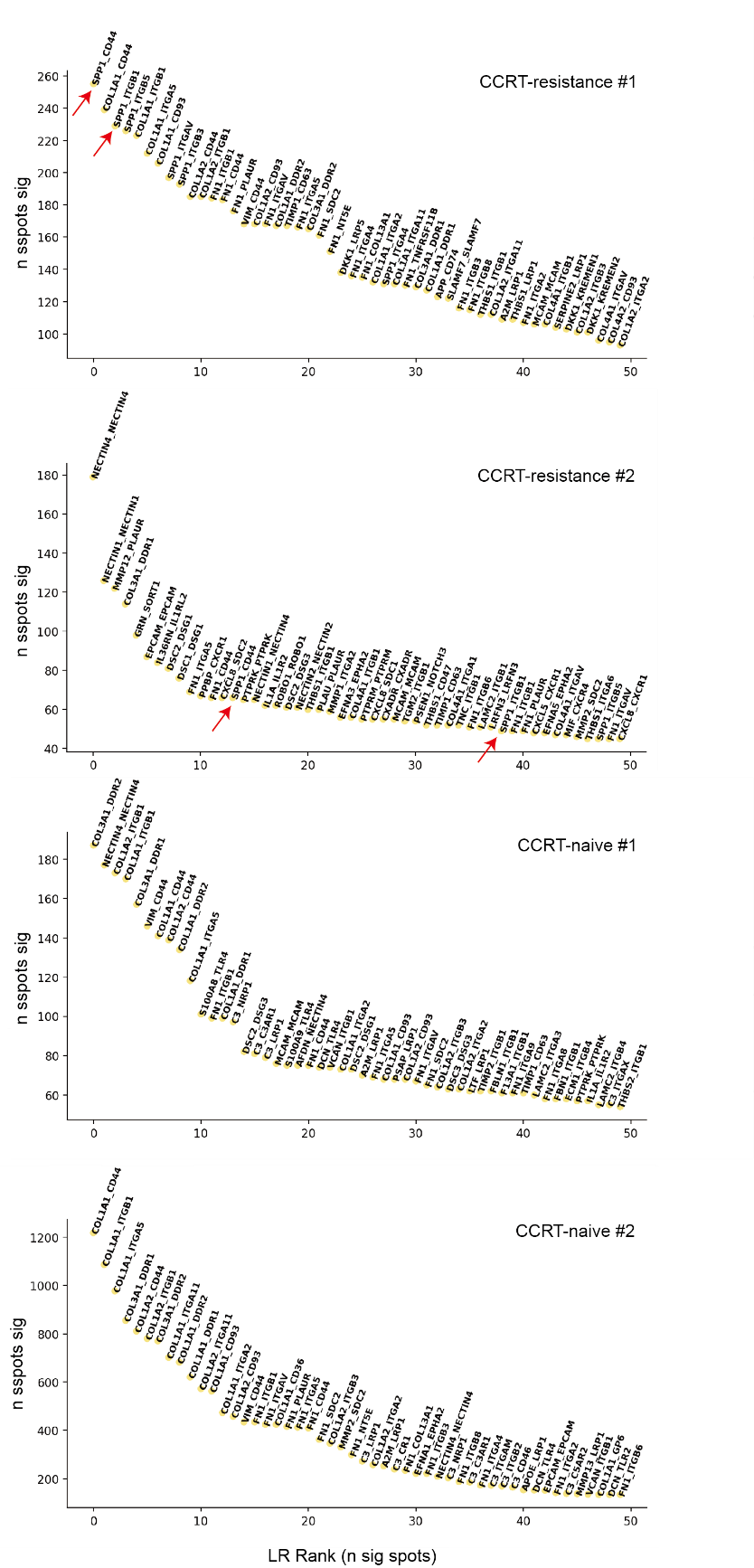

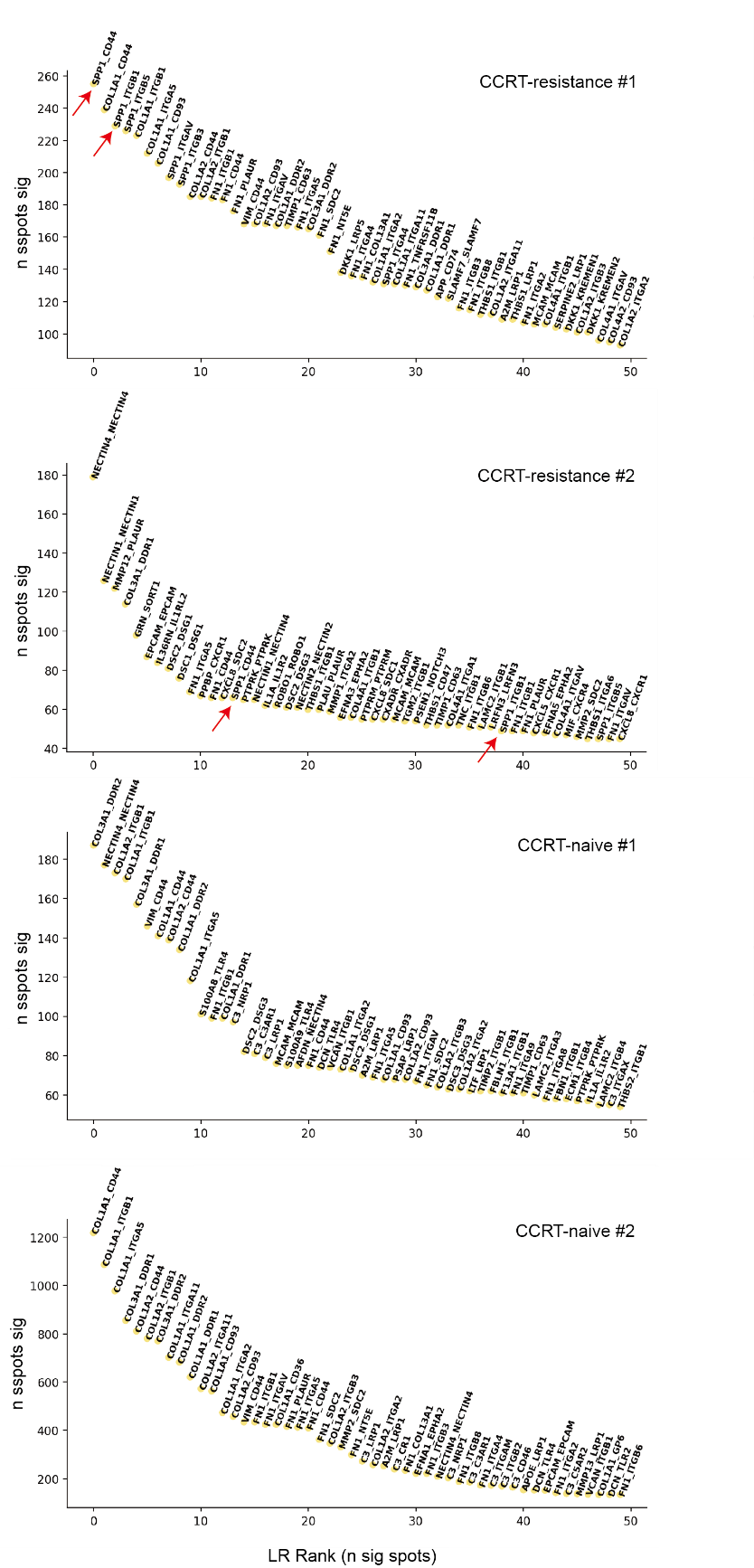


Supplementary Figure 2. The top 50 LR interaction pairs were identified using the *stLearn* tool, which incorporates spatial location and co-expression in the ST data to infer the number of spots with significant interactions. In CCRT-resistance samples, *SPP1* showed a high interaction with *CD44* and *ITGB1* (indicated by red arrow).

Abbreviation: CCRT, concurrent chemo-radiotherapy; LR, ligand-receptor; ST, spatial transcriptome


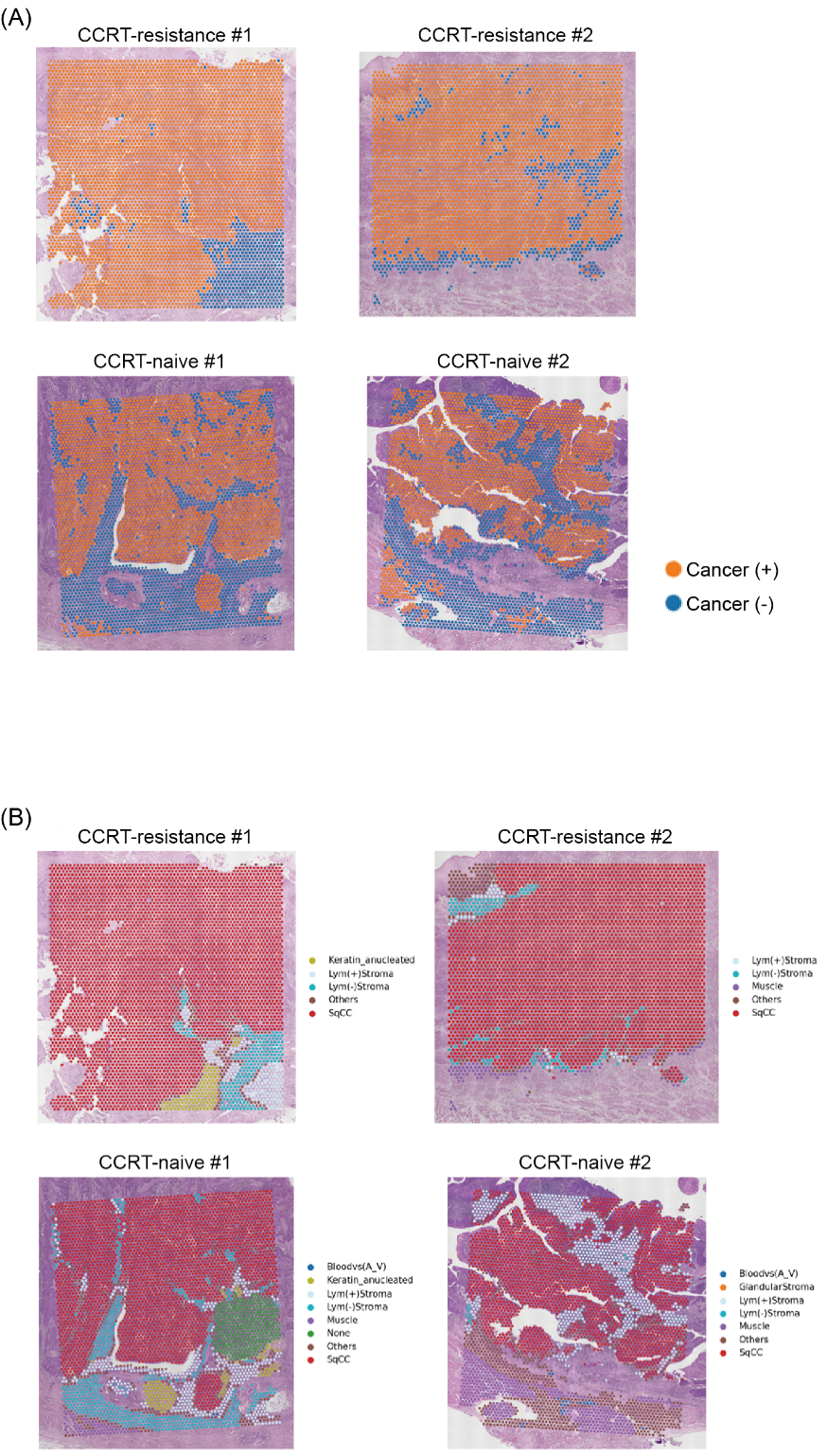

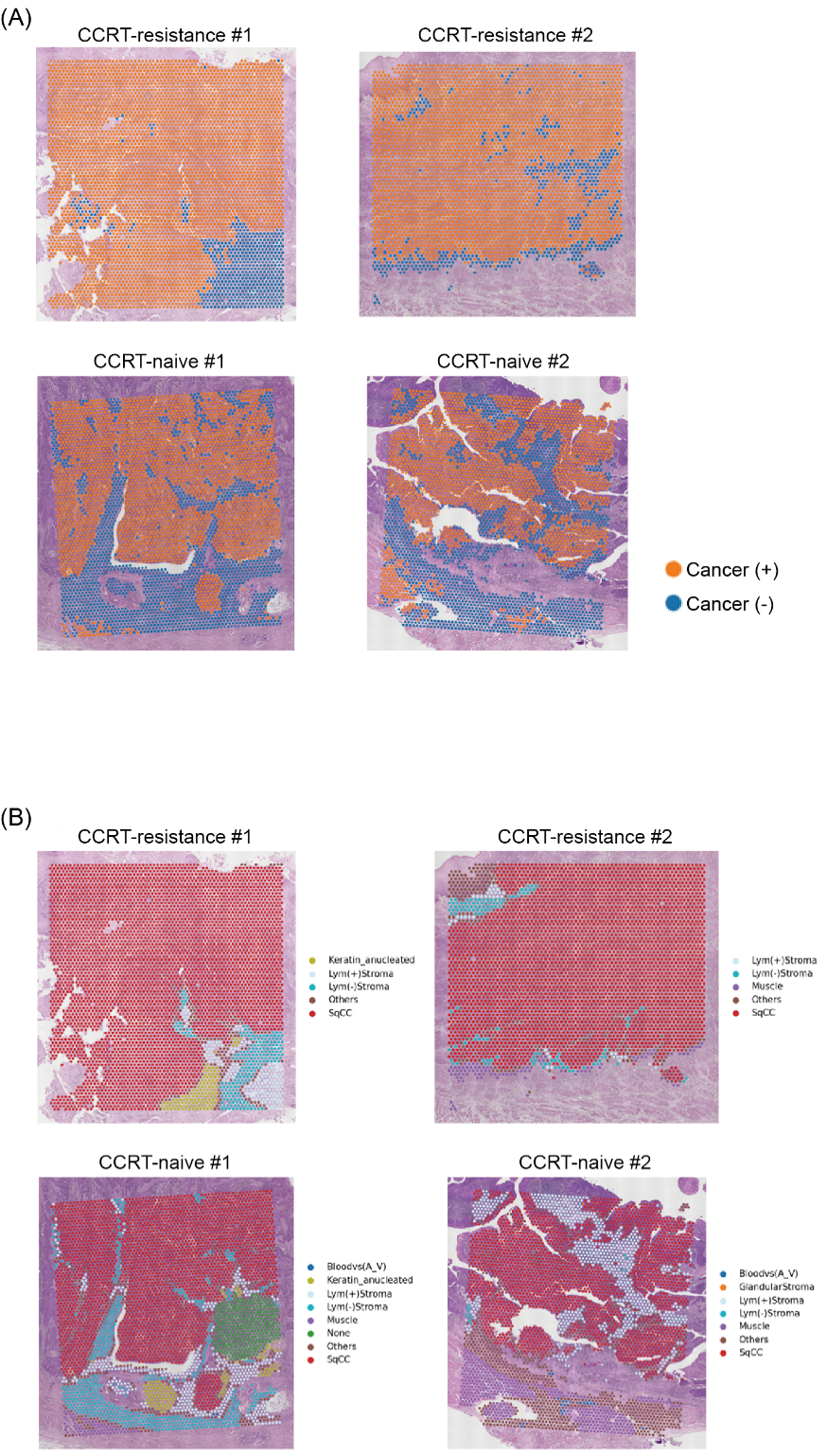


Supplementary Figure 3. Annotation of cancerous and non-cancerous regions. (A) Application of the *Cancer-Finder* algorithm to four hypopharyngeal SCC ST datasets (B) Annotation by a board-certified pathologist. The annotated spots were overlaid on the H&E-stained slides (refer to Annotation of the cancer region and calculation of distance from the cancer in MATERIALS AND METHODS)

Abbreviation: SCC, squamous cell carcinoma; ST, spatial transcriptomics


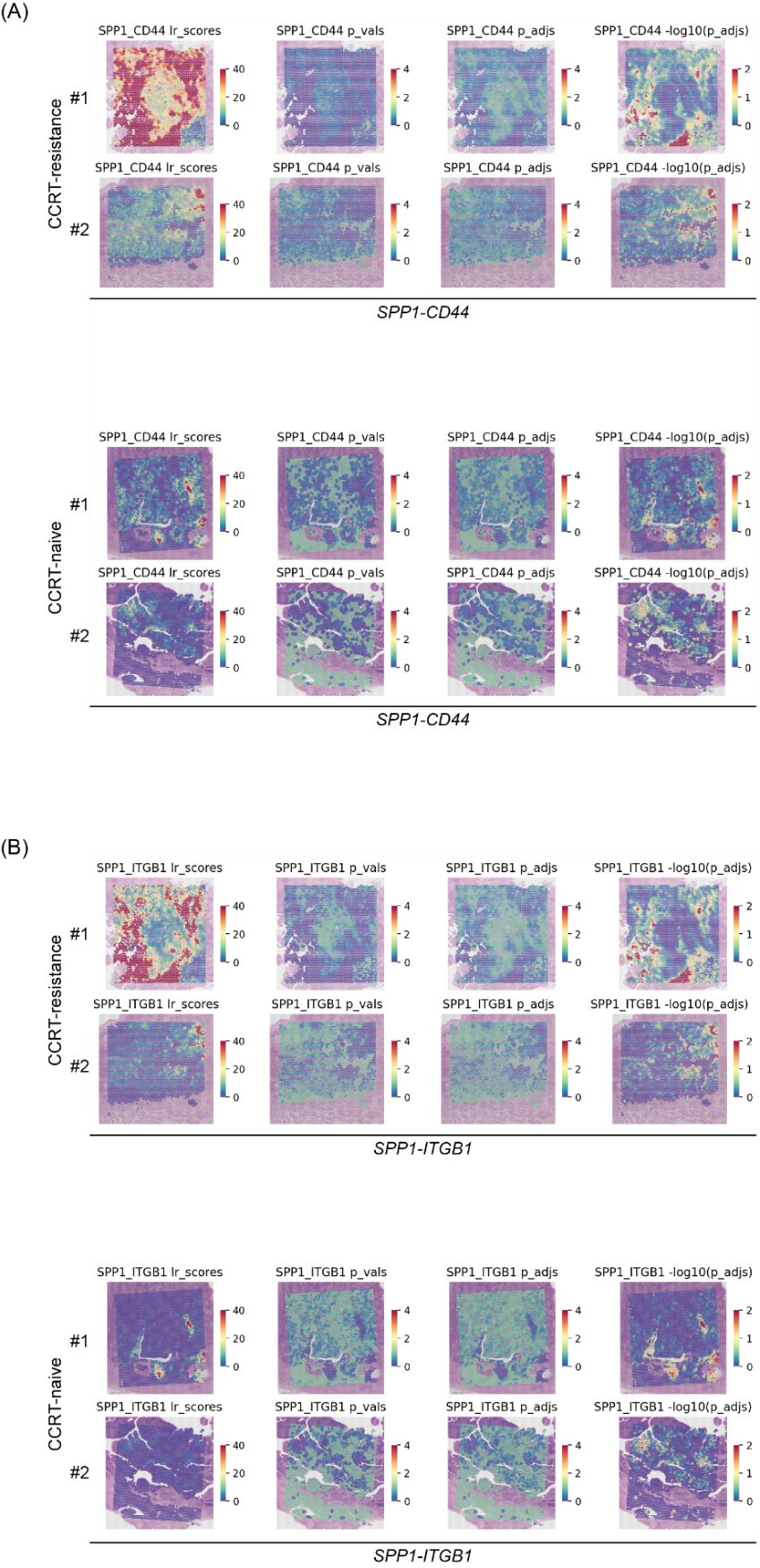

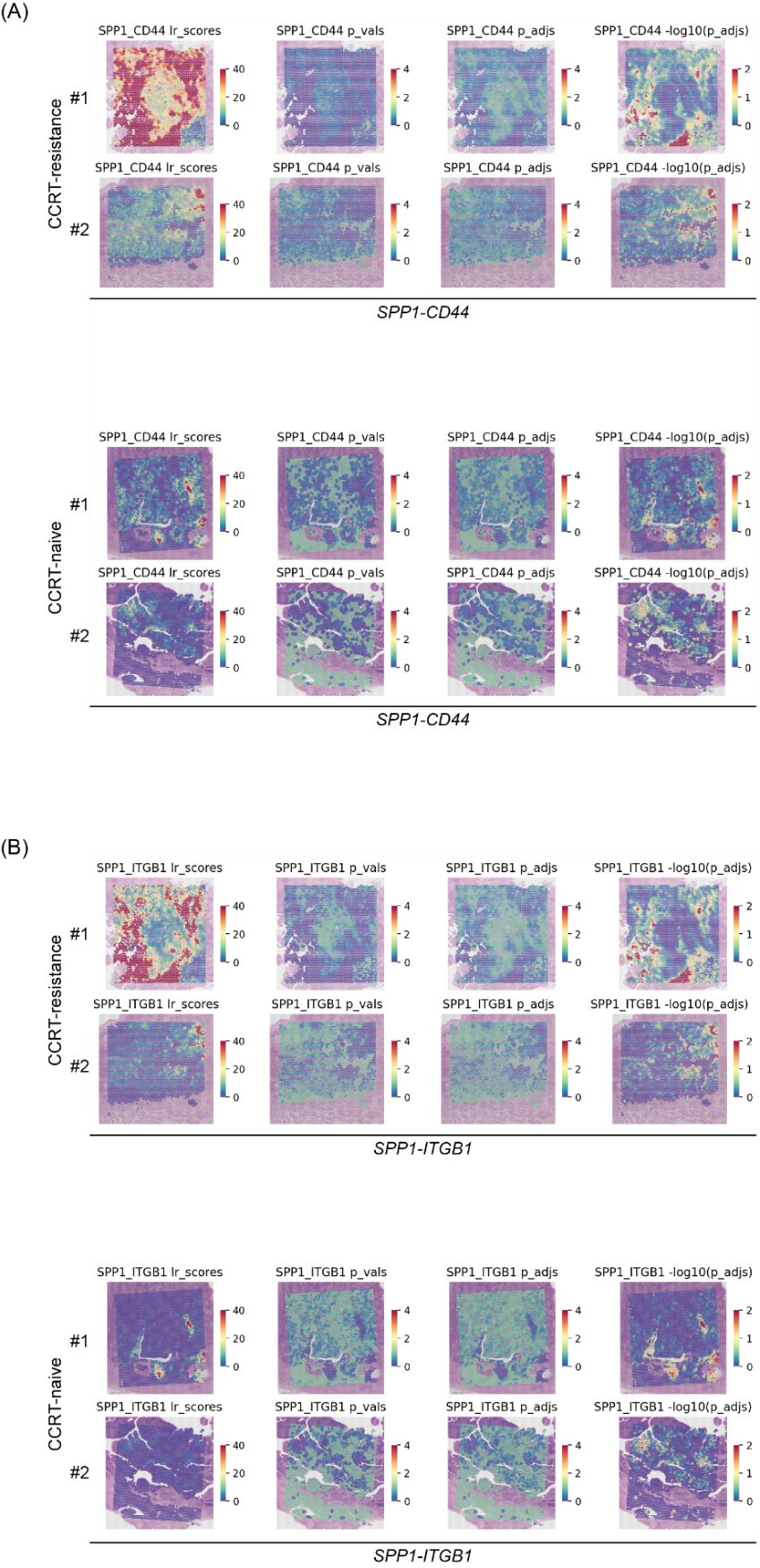


Supplementary Figure 4. Spatial heatmap for indexes of LR interactions for *SPP1-CD44* (A) and *SPP1-ITGB1* (B), overlaid on an H&E-stained slide image in each sample. “lr_scores” represent the strength of ligand-receptor colocalization, “p_vals” indicate the significance of “lr_scores” determined by creating random distribution of non-interacting gene-gene pairs, and “p_adjs” show the adjusted p values.

Abbreviation: LR, ligand-receptor


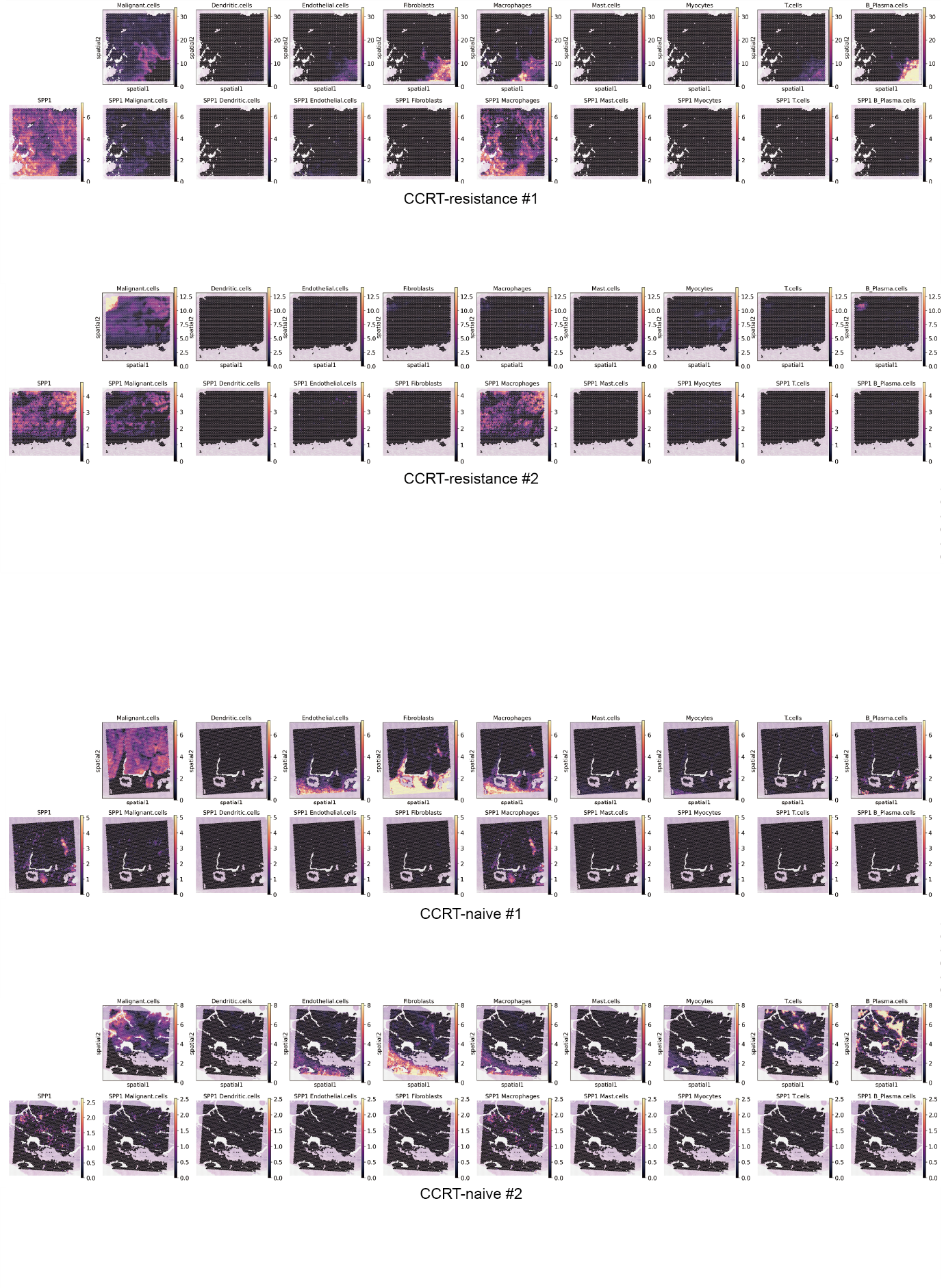

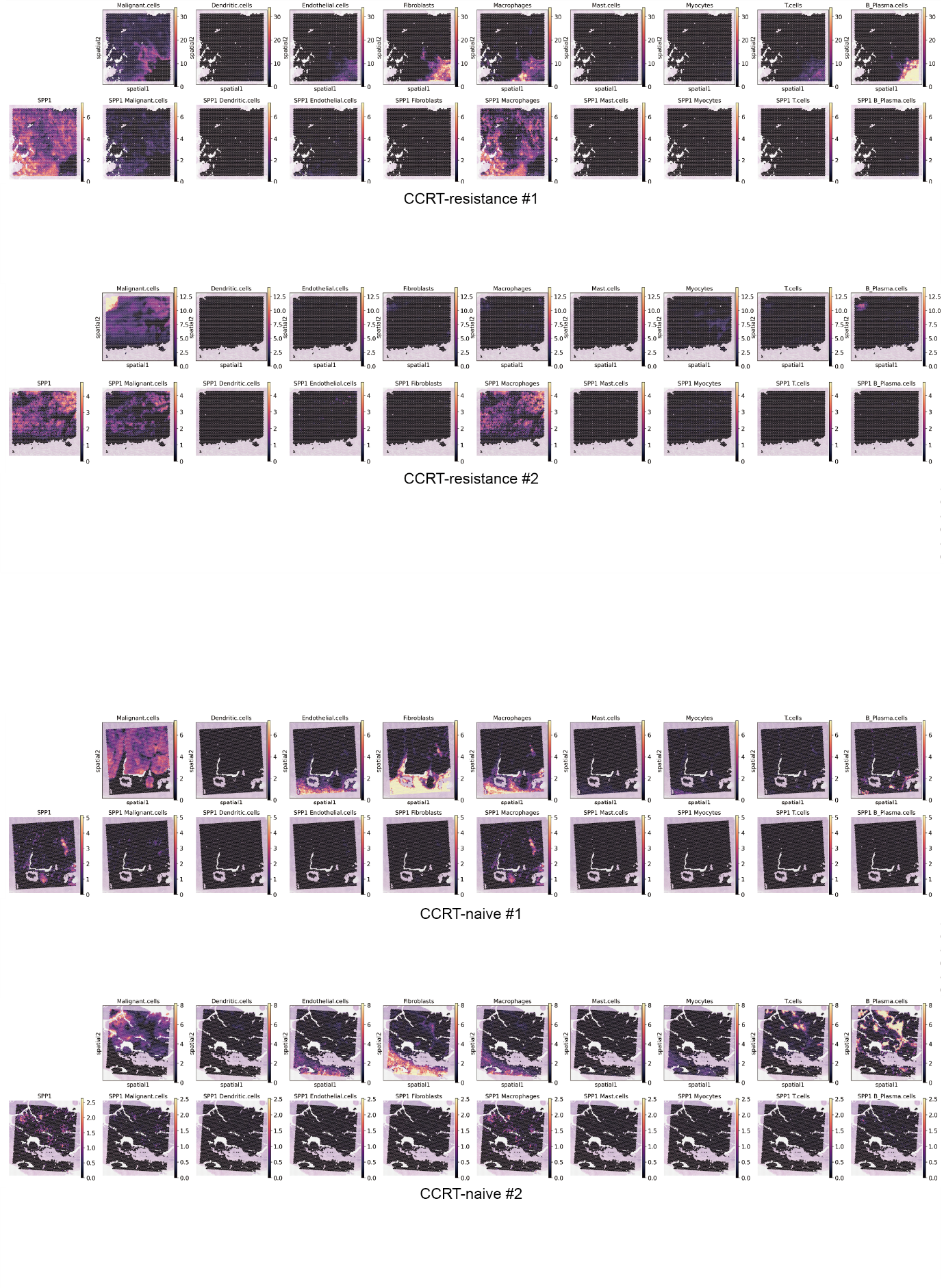


Supplementary Figure 5. Heatmaps showing the abundance of malignant epithelial cells and other cell types comprising the TME (dendritic cells, endothelial cells, fibroblasts, macrophages, mast cells, myocytes, T cells, and plasma cells) on ST spots for each hypopharyngeal SCC sample (top row of each panel). The bottom row of each panel illustrates the spatial expression of *SPP1* and cell type-specific *SPP1* expression in four hypopharyngeal SCC samples. This figure was generated using the *Cell2location* package.

Abbreviation: CCRT, concurrent chemo-radiotherapy; SCC, squamous cell carcinoma; ST, spatial transcriptomics; TME, tumor microenvironment


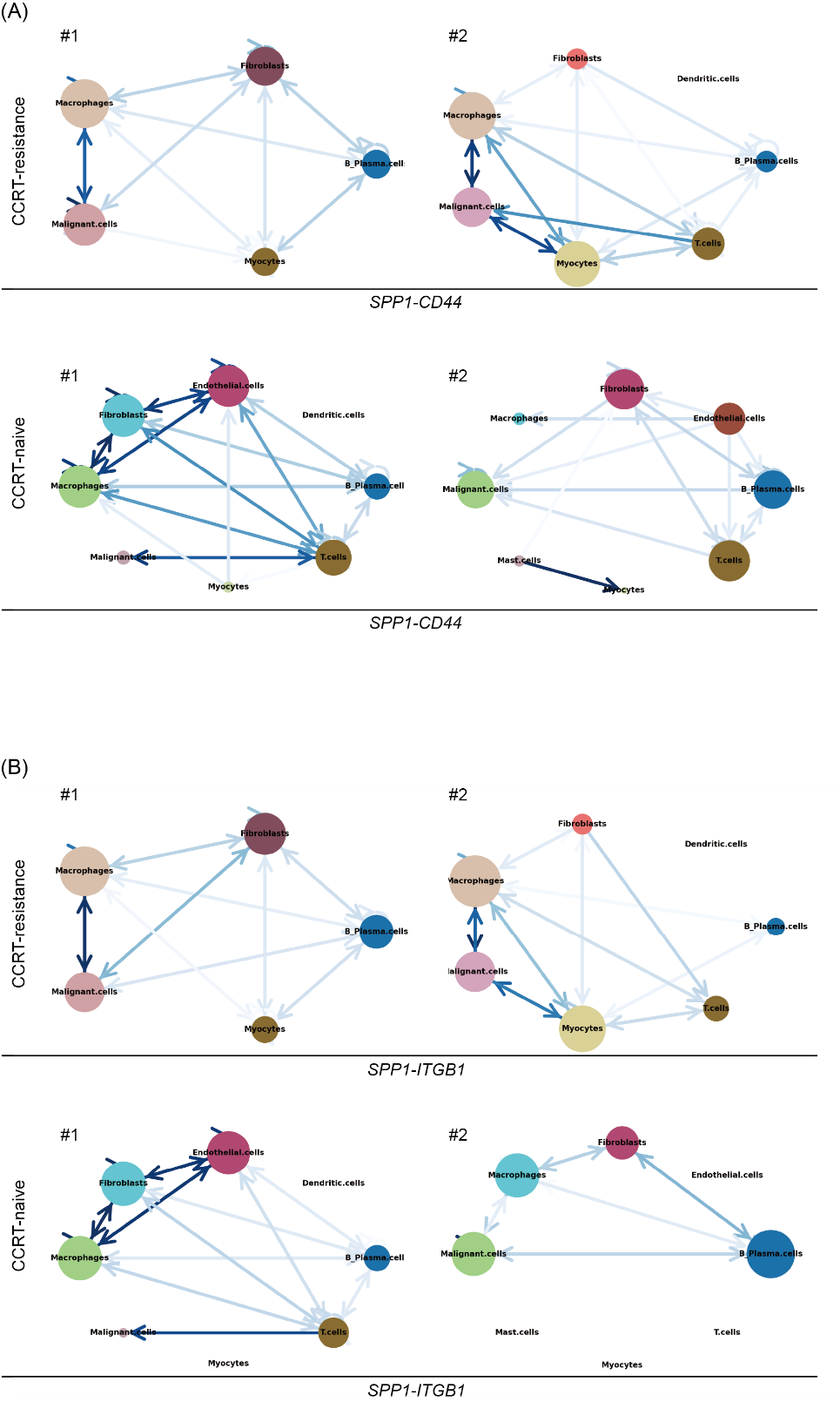

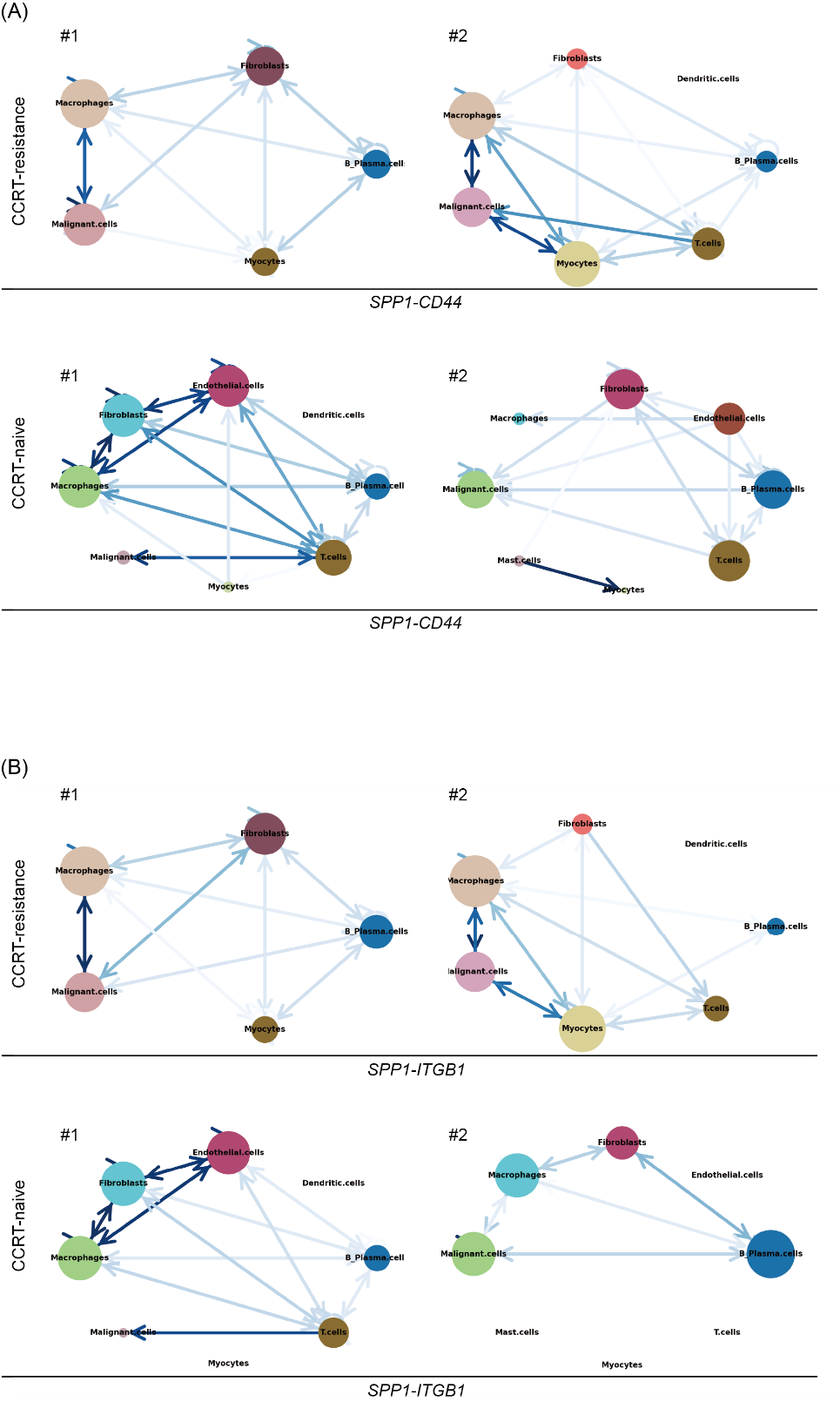


Supplementary Figure 6. In each study samples, cell-cell interactions were analyzed to identify predominant cells involved in LR interactions, *SPP1-CD44* (A) and *SPP1-ITGB1* (B). The size of the spots represents the total number of significant spot interactions the cell type is involved in, and the color of the edge represents the number of significant interactions between the two cell types. In CCRT-resistance samples, *SPP1-CD44* and *SPP1-ITGB1* interactions occur primarily between malignant epithelial cells and macrophages, while these interactions occur mainly between macrophages and endothelial cells or fibroblasts in CCRT-naive samples.

Abbreviation: CCRT, concurrent chemo-radiotherapy; LR, ligand-receptor

Supplementary File

Top 20 gene markers for the annotation of each spot cluster.
